# GaTO: An Ontological Model to Apply Gamification in Intelligent Tutoring Systems

**DOI:** 10.3389/frai.2019.00013

**Published:** 2019-07-31

**Authors:** Diego Dermeval, Josmário Albuquerque, Ig Ibert Bittencourt, Seiji Isotani, Alan Pedro Silva, Julita Vassileva

**Affiliations:** ^1^Faculty of Medicine, Center of Excellence for Social Technologies, Federal University of Alagoas, Maceió, Brazil; ^2^Center of Excellence for Social Technologies, Computing Institute, Federal University of Alagoas, Maceió, Brazil; ^3^Department of Computing Systems, Institute of Mathematics and Computacional Sciences, University of São Paulo, São Carlos, Brazil; ^4^Department of Computer Science, University of Saskatchewan, Saskatoon, SK, Canada

**Keywords:** intelligent tutoring systems, gamification, gamified intelligent tutoring systems, ontologies, ontology-aware authoring tools

## Abstract

Intelligent Tutoring Systems (ITSs) are concerned with the use of artificial intelligence techniques for performing adaptive tutoring to learners' according to what they know about the domain. Researchers are increasingly interested in applying gamification in e-learning systems to engage students and to drive desired learning behaviors. However, little attention has been drawn to the effective application of gamification in ITS, and how to connect theories of both concepts in a standard and formal way. Moreover, gamified ITS should manipulate a huge amount of knowledge regarding several models, i.e., gamification, domain, student and pedagogical models. Formally connecting such theories as well as representing system's knowledge relies on the use of ontologies. In this paper, we present an ontological model that connects gamification and ITS concepts. Our model takes advantage of ontologies to allow automated reasoning (e.g., on the domain, student, pedagogical or gamification models), to enable interoperability, and create awareness about theories and good practices for the designers of gamified ITS. To evaluate our model, we use an ontology evaluation method based on five knowledge representation roles. We also illustrate how it could support the development of an intelligent authoring tool to design gamified ITS.

## 1. Introduction

Intelligent tutoring Systems (ITSs) have been drawing the attention of academics and practitioners since early 70's (Woolf, 2010). ITSs deserve special attention since we can find strong empirical evidence that in some situations they can successfully complement and substitute other instructional models, and that these situations exist at all educational levels and in many common academic subjects (Ma et al., 2014). However, during the learning process by using ITSs, it is very common that students become disengaged or bored (Jackson and McNamara, 2013). By contrast, motivated, challenged and intrigued students tend to have better learning results (VanLehn, 2011). In this way, relying on theories and models of motivation and human behavior, many works have been using persuasive technologies, for instance, gamification, to address the students' disengagement and lack of motivation problems (Hamari et al., 2014).

Deterding et al. (2011) define gamification as the use of game design elements in non-game contexts. These contexts (e.g., education, e-commerce, healthcare, and so on) mostly converges to a common final objective, the use of gamification to engage and motivate users to achieve better results and create enhanced solutions and experiences (Hamari et al., 2014). In the educational context, gamification may motivate action, promote learning, and facilitate problem solving (Seaborn and Fels, 2015) as well as drive desired learning behaviors (Kapp, 2012).

To explicitly consider motivational aspects, researchers have been addressing the use of gamification along with ITS (González et al., 2014; Andrade et al., 2016; Dermeval, 2016; Shi and Cristea, 2016; Dermeval et al., 2018). The design of gamified ITS should include the development of four classic components (i.e., domain, student, tutoring, and interface) as well as a gamification model. However, the application of gamification in ITS must deal with the connection of theories from both topics. Moreover, ITSs are knowledge-intensive systems that handle knowledge about the domain of the tutor, students' behaviors, tutoring theories, and so on. The inclusion of gamification generates additional knowledge about gamification elements, good design principles for using gamification, students' motivation, etc.

In the meantime, there is a growing interest in the use of ontologies to address e-learning problems. Particularly, in the context of ITS, ontologies have been used to represent the domain model concepts, to represent students' modeling allowing automated reasoning, to interoperate heterogeneous ITSs, and so on (Al-Yahya et al., 2015). Ontology is defined as “explicit specification of a conceptualization” (Gruber, 1993). It is “explicit” because of its classes and properties visibility. Conceptualization is understood to be an abstract and simplified version of the world to be represented. Moreover, ontologies can be logically reasoned and shared within a specific domain (Guarino, 1998). Thus, ontologies are a standard form for representing the concepts within a domain, as well as the relationships between those concepts in a way that allows automated reasoning.

Hence, formally representing gamification and ITS concepts could provide several benefits to the design of gamified intelligent tutoring systems. It could allow the automated reasoning off all knowledge manipulated by these systems, which could also favor machines to automatically handle it. It might also provide a standard representation for the infrastructure of gamified ITSs, which may enable the interoperability (e.g., to interoperate educational resources) between different architectures of these systems. Furthermore, it may also leverage the transparency of the theories used to design these systems as well as allowing representing good design principles for effectively designing gamified ITS—i.e., the later benefits could be very useful to aid the design of authoring tools for constructing such systems.

In this paper, we present the GaTO ontological model, which connects gamification concepts and theories to ITS' concepts of the domain, student and pedagogical models. In order to develop our ontological model, we used the METHONTOLOGY approach, which is an ontology engineering methodology that is divided into seven main phases (Gómez-Pérez, 1996; Fernández-López et al., 1997). Our decision on such methodology was made since it is listed as one of the most mature ontology engineering methodologies existing in literature. Moreover, it includes activities to support most activities of the ontology development lifecycle (Corcho et al., 2003; Bautista-Zambrana, 2015). To evaluate our model, we use an ontology evaluation method that is based on knowledge representation roles (Davis, 1997), i.e., Substitute, Ontological Commitment, Intelligent Reasoning, Computational Efficiency, and Human Expression. We also illustrate how our ontological model could be used to support the development of a theory and evidence-aware authoring tool to design gamified ITS.

This paper is organized as follows. Section 2 presents our ontological model describing its background and how we link gamification and ITS concepts. Then, in section 3, we describe how we evaluate our ontological model. Section 4 demonstrates the usefulness of our ontological model by illustrating how such model can be used to develop intelligent authoring tools that enable the design of gamified ITSs. Then, section 5 presents related works on the use of ontologies to model gamification and intelligent tutoring systems. Finally, in section 6, we point out the conclusion of our work and recommendations for future research.

## 2. Ontological Model: Gamification Domain Ontologies and Gamified Intelligent Tutoring Ontology

Gamification is an emerging topic with several concepts, theories, and definitions. Thus, during our ontological model engineering process, we decided to represent core concepts (e.g., gamification definition, game design element, player model, and so on) regarding gamification domain and specific gamification concepts (e.g., gamification design framework, gamification design practices, specific player models, and so on) in two different ontologies in our model. In this way, as we are representing concepts concerning the gamification domain, we developed a domain ontology to represent these concepts.

**Figure 1:**
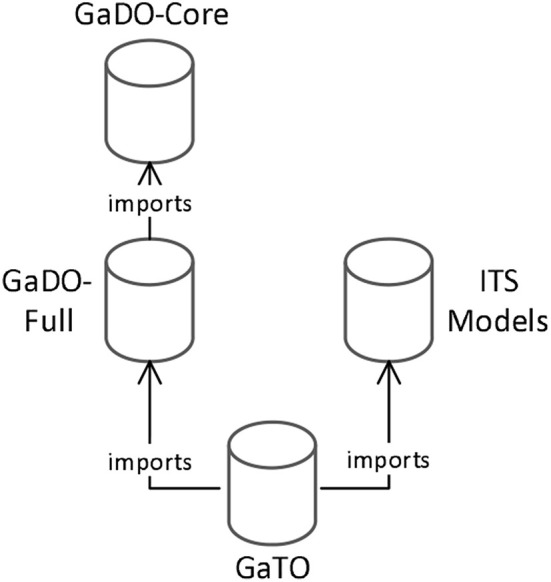
Ontological model illustrating the relationship between gamification and ITS ontologies.

In the following sections, we present how we developed the Gamification Domain Ontology (GaDO) (section 2.2) including the two sub-domain ontologies: GaDO-core and GaDO-full. Next, in section 2.3, we present how we specified an additional ontology that indeed connects the concepts of these ontologies with ITS concepts, called Gamification Tutoring Ontology (GaTO). Figure 1 presents an overview of the ontologies illustrating how they are related to each other.

In order to develop these ontologies, we used the METHONTOLOGY approach, which is an ontology engineering methodology that is divided into seven main phases (Gómez-Pérez, 1996; Fernández-López et al., 1997), as explained in the next section. Our decision on such methodology was made since it is listed as one of the most mature ontology engineering methodologies existing in the literature. Moreover, it includes activities to support most activities of the ontology development life-cycle (Corcho et al., 2003; Bautista-Zambrana, 2015).

### 2.1. Methontology

METHONTOLOGY is a methodology that describes a set of phases and techniques to build an ontology either from scratch or by reusing other ontologies. The ontology development process by using this methodology identifies the required tasks when working on an ontology, i.e., planification, specification, knowledge elicitation, conceptualization, formalization, integration, implementation, evaluation, documentation, and maintenance. With the ontology life-cycle, these tasks acquire order and depth through the ontology lifetime. Therefore, the methodology framework was built based on these concepts, specifying the used techniques, determining which products are obtained, and deciding how to evaluate each activity.

### 2.2. Gamification Domain Ontology (GaDO)

The purpose of this ontology is modeling the main gamification concepts in order to support its application in intelligent tutoring systems. As previously mentioned, this domain ontology is divided into two sub-domain ontologies, which are described in this section.

#### 2.2.1. Gamification Domain Ontology (GaDO) – Core

As explained in section 2.1, the first step of the METHONTOLOGY is defining the specification step. In the specification step for this ontology, we first defined its scope. It mainly considers core concepts regarding the gamification definition, which includes, for example, game design element and context. It involves players, player model and player type abstract concepts regarding a specific gamified context. We also specify concepts regarding abstract theories of motivation and needs that are supporting gamification.

Our main sources for knowledge acquisition include the works by Deterding et al. (2011), Werbach and Hunter (2012), and Hamari et al. (2014) in order to specify gamification concepts according to the definition provided by these authors. We also relied on three systematic literature reviews—i.e., Hamari et al. (2014), Seaborn and Fels (2015), and de Sousa Borges et al. (2014)—that, as previously mentioned, summarize a plethora of studies that use gamification in several contexts. For each systematic literature review, we consider the whole list of papers included in it as sources of knowledge to conceptualize our ontology. In addition, we also take into account the work by Challco et al. (2014) since it presents an ontology that conceptualizes gamification to be applied in a specific kind of educational system, i.e., computer-supported collaborative learning (CSCL).

Next, following the METHONTOLOGY process, we performed the conceptualization of our ontology. This phase includes defining the core concepts, a glossary of terms, a tree of concepts, and binary-relations between the concepts in the ontology. Based on our sources of knowledge, we defined the following core concepts: Gamification, Game Design Element, Context, Motivation and Need Theory, Player, Player Model, and Player Type.

The next phase includes integrating the conceptualization with existing ontologies on the topic. However, we could not find any other gamification domain ontology that could be reused in our ontological model. One potential ontology for reuse is the one presented by Challco et al. (2014), however, although that work has been considered a source of knowledge for our ontological model, it is particularly tied to the context of CSCL. Thus, we could not reuse such ontology in our domain ontology.

**Figure 2:**
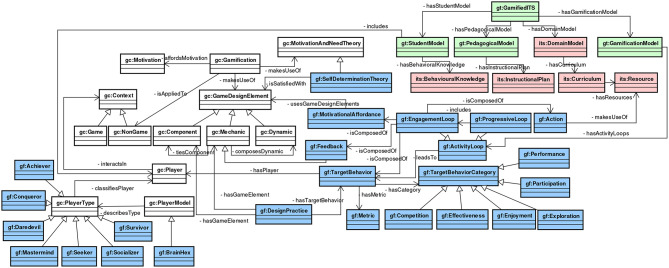
Excerpt of the Gamification Tutoring Ontology (GaTO). Some classes and relations are omitted for clarity. The prefix “gc” and “gf” refer to the concepts of the GaDO-core and GaDO-full ontologies, respectively. The prefix “gt” refers to the GaTO concepts and “its” refers to concepts from Bittencourt's ontology.

In the implementation phase, we implemented the GaDO-core ontology in a RDF/OWL file^1^ with the aid of Protégé tool. Figure 2 presents (page 12) an excerpt of this ontology integrated with the other ontologies. Ignore for now the blue, green and red classes. In the sequel, we explain each of its concepts and relations.

Based on the gamification definition provided by the sources of knowledge we considered, we linked the concept of *Gamification* with several core concepts of this ontology. As seen in Figure 2, *Gamification* can rely on a set of *Motivational and Need Theories* in order to afford motivation. Following its definition, it is applied to a non-game context and also makes use of different types of *Game Design Elements*, which can be one of three types: *Dynamic, Mechanic*, and *Component*. According to Werbach and Hunter (2012), each one of these types can be specialized in several other elements; the subtypes do not appear on the figure due to lack of space. *Dynamic* can be one of the following types: *Constraints, Emotions, Narrative, Progression*, and *Relationships*. In turn, *Mechanics* can be *Challenges, Chances, Competition, Cooperation, Feedback, Resource Acquisition, Rewards, Status, Story, Theme, Transactions, Turns*, and *Win States*. The *Component* type can also be sub-specialized in several types: *Achievements, Avatars, Badges, Boss Fights, Collections, Combat, Content Unlocking, Gifting, Leaderboard, Levels, Points, Quests, Social Graph, Team, Time Constraint*, and *Virtual Goods*. Another important concept of this ontology is the *Player*, which interacts in a particular context that can be *Game* or *Non-Game*. A *Player* is classified by a *Player Type*, whereas a *Player Type* is described by a *Player Model*.

The documentation of this ontology^2^ was produced throughout the execution of all previous phases. Finally, in the last phase, we evaluate the generated ontology. However, as METHONTOLOGY does not explicitly define how to evaluate ontologies generated using such methodology, we choose our own strategy according to the existing works on ontologies evaluation. We explain how we evaluate this ontology as well as the other ontologies presented in this section in section 3.

#### 2.2.2. Gamification Domain Ontology (GaDO) – Full

In a similar way to the development of the GaDO-core ontology, we followed the METHONTOLOGY process steps for conceptualizing the GaDO-full ontology. As such, we first defined the scope of GaDO-full, which mainly considers a particular theory of motivation (i.e., Self- Determination Theory), a player model (i.e., BrainHex), and a gamification design framework (e.g., 6D framework) as well as how these concepts are linked to GaDO-core concepts. We also consider in the scope of this ontology the idea of gamification design practice, which is a pre-designed set of gamification elements linked to specific target behaviors that could be further used to aid the design of gamified ITS.

As previously mentioned, there are many game design elements (e.g., points, badges, levels, leaderboard, etc.) that could be used along with educational systems. Researchers are increasingly investigating the effects of gamification at several application contexts, including education (Nacke and Deterding, 2017). In fact, identifying which game design elements effectively benefit learning performance as well as motivation and engagement of students is still an open issue. For instance, several works included in systematic literature reviews (de Sousa Borges et al., 2014; Hamari et al., 2014; Seaborn and Fels, 2015) present combinations of game design elements that might be more amenable to effectively achieve particular behaviors. As such, to identify which game design elements combinations might be effective for learners in the e-learning context, we analyze the empirical works that provide evidence for using particular combinations of game design elements to target specific behaviors in the e-learning domain.

To analyze the empirical works included in the reviews with respect to educational contexts, we use the framework proposed by Hamari et al. (2014). This framework conceptualizes gamification as a process which includes motivational affordances, psychological outcomes and behavioral outcomes. According to this conceptualization, gamification is defined as a process of enhancing services with (motivational) affordances in order to invoke gameful experiences (psychological outcomes) and further behavioral outcomes. Thus, for each paper that present empirical evidence on the effect of using game design elements (motivational affordances) to target behavioral outcomes (e.g., improving learning outcomes, increasing engagement, and so on etc.), we used Hamari's framework to classify it.

Based on the classification of game design elements and behavioral outcomes the elements help to achieve, we group the effects of these elements by behavioral outcomes. Thus, we identified six main behavioral outcomes achieved by the use of gamification in the studies: participation, performance, enjoyment, exploration, competition and effectiveness.

We summarize the target behaviors we identified along with the game design elements that might help to achieve them based on the works analyzed in Table 1. The mapping of behavioral outcomes and motivation affordances (i.e., game design elements) is used to constrain the design space of gamified ITS considering empirical studies on the topic as well as used in the conceptualization of a gamification domain ontology, as presented in the following sections. The behaviors are described on below:

- Participation: this behavior includes game design elements that are more amenable to increase the level of participation/engagement of students based on the results provided by Denny (2013), Halan et al. (2010), Fitz-Walter et al. (2012), Spence et al. (2012), Domínguez et al. (2013), Li et al. (2012), Foster et al. (2012), Goehle (2013), and Snyder and Hartig (2013). It may include the following elements: *Challenge, Levels, Leaderboard, Story, Badges, Rewards*, and *Points*;
- Performance: this behavior includes game design elements that were used by several works (Smith and Baker, 2011; Cheong et al., 2013; Domínguez et al., 2013; Hakulinen et al., 2013) suggesting their use for increasing students' learning outcomes. It includes the following elements: *Story, Feedback, Rewards, Badges, Challenges, Leaderboard, Points*, and *Levels*;
- Enjoyment: this behavior encompasses the game design elements used in the empirical works that are amenable to increase students' enjoyment (i.e., fun) (Landers and Callan, 2011; Hernández Ibáñez and Barneche Naya, 2012; Li et al., 2012; Denny, 2013). The following game design elements are included in this behavior: *Story, Rewards, Badges, Points, Avatar*, and *Challenges*;
- Exploration: this behavior is supported by some empirical works (Fitz-Walter et al., 2011; Spence et al., 2012) which suggest that using some game design elements could enhance the exploration of the educational system by students. The following elements are included within this category: *Levels, Challenge*, and *Boss fight*;
- Competition: this behavior is suggested by the results provided by Domínguez et al. (2013). Using *Leaderboard* and *Points* may enhance competition between students, which we define as *Competition* behavior;
- Effectiveness: we also defined an additional target behavior based on Domínguez et al. (2013), which we call *Effectiveness* behavior. This behavior suggests that using *Leaderboard, Badges, Points* there might be an increase in students' effectiveness while they interact with the educational system.

**Table 1:** Summary of target behaviors and game design elements.

**Target behavioral outcome**	**Game design elements**
Participation	Story, rewards, badges, levels, challenge, leaderboard, points
Performance	Story, feedback, rewards, badges, levels, challenge, leaderboard, points
Competition	Leaderboard, points
Enjoyment	Story, rewards, badges, avatars, challenge, points
Exploration	Levels, challenge, boss fight
Effectiveness	Leaderboard, badges, points

Our main sources for knowledge acquisition include the works by Werbach and Hunter (2012) and Deci and Ryan (2010) in order to link the Self-determination theory concepts to GaDO-core concepts. We also relied on the work by Nacke et al. (2014) to specify the BrainHex player model along with its seven-player types. As gamification design framework, we chose the 6D framework since it is based on the Self-Determination Theory (Werbach and Hunter, 2012) and is the more comprehensive available gamification framework in the literature (Mora et al., 2015). Thus, these references were also used as sources of knowledge to link the 6D framework to GaDO-core concepts. Additionally, we also relied on the systematic literature reviews (de Sousa Borges et al., 2014; Hamari et al., 2014; Seaborn and Fels, 2015) as well as on the empirical papers listed in the reviews on the use of gamification in education to specify the concept of gamification design practice for the education context. As previously described, this concept is further used in the gamified tutoring ontology to constrain the gamification design space linking target behaviors to particular sets of game design elements based on the pieces of evidence provided by the empirical studies.

Next, we performed the conceptualization of this ontology. Likewise GaDO-core conceptualization, in this phase we define the core concepts, a glossary of terms, a tree of concepts, and binary-relations between the concepts in the ontology. Based on our sources of knowledge, we defined the following core concepts: Self-Determination Theory, Activity Loop, Engagement Loop, Motivational Affordance, Feedback, Target Behavior, Metric, Design Practice, and BrainHex Model.

As previously explained, the GaDO-full ontology makes use of the GaDO-core ontology to specialize particular concepts we are considering. In this way, in the integration phase of this ontology, we import the GaDO-core ontology in order to integrate this ontology's concepts to GaDO-core concepts. We could not find any other ontology that could be integrated to our ontology.

In the implementation phase, we also implemented the GaDO-full ontology in an RDF/OWL file^3^ with the aid of Protégé tool. Figure 2 presents the ontological model integrated with GaDO-core and GaTO (which will be further explained) in a UML conceptual model—the blue classes represent the concepts of the GaDO-full ontology—ignore for now the red and green classes, as they will be explained in the next section. In the sequel, we explain GaDO-full concepts and relations as well as how they are integrated with GaDO-Core ontology.

The main concepts of GaDO-full ontology are related to the 6D framework components and how they are connected to GaDO-core ontology. This framework is supported by the *Self-Determination Theory*, which is represented in this ontology as a specialization of the *Motivation and Need Theory*, as shown in Figure 2. Werbach and Hunter (2012) establish that this framework has six steps: (i) Define business objectives; (ii) Delineate target behavior; (iii) Describe your players; (iv) Devise activity loops; (v) Don't forget the fun; and (vi) Deploy appropriate tools. Recall that our ultimate goal (which is not necessarily in the scope of this work) is to apply gamification to intelligent tutoring systems in order to increase engagement and motivation of students of these systems, expecting to increase their learning performance. Hence, this is the main general objective of this work. Indeed, only steps (ii), (iii), and (iv) are in the scope of this ontology conceptualization, since, the last two steps—i.e., (v) and (vi) may be only satisfied through the implementation of gamified intelligent tutoring systems. For instance, to not forget the fun it might be needed to investigate several aspects of the gamification design (components, mechanics, and dynamics game design elements).

As seen in Figure 2, a *Target Behavior* has a category (*TargetBehaviorCategory* class) and a success *Metric*. A *Target Behavior Category* can be one of the following types that we identified in Table 1: *Performance, Participation, Exploration, Enjoyment, Effectiveness*, and *Competition*. Although not explicitly presented in Figure 2, since the specializations of *Component* and *Mechanic* game design elements are suppressed for simplicity purpose, the design elements summarized in Table 1 are directly related (using object properties) to their correspondent target behavior category in the ontology.

Regarding activity loops (*ActivityLoop* class), its implementation intends to lead to particular target behaviors and they can be of two types: *Engagement Loop* and *Progressive Loop*. According to Werbach and Hunter (2012), an *Engagement Loop* is composed of three components: motivation, action, and feedback. In our conceptualization, motivation is represented by the use of *Motivational Affordances*, which are related to *Game Design Elements*, whereas *Feedback* is a *Mechanic* game design element. The *Action* component is connected to ITS concepts, since the interaction of the student in the tutor will occur with resources provided by it, as will be further explained in the GaTO ontology (section 2.3). Furthermore, an *Engagement Loop* is also related to a *Target Behavior*, which in turn is related to a particular *Player*. Moreover, a *Progressive Loop* includes the gamification design to drive different levels of gamification, thus, in our conceptualization we consider that it includes a set of *Engagement Loops* for each level. We also specify the *BrainHex* player model as a specialization of *Player Model* as well as its *Player Types*: *Achiever, Conqueror, Daredevil, Mastermind, Seeker, Socializer* and *Survivor* (Nacke et al., 2014).

Likewise GaDO-Core ontology, the documentation of this ontology^4^ was produced throughout the execution of all previous phases. Finally, in the last phase, we evaluate the generated ontology, as will be further explained in section 3.

### 2.3. Gamified Intelligent Tutoring Ontology (GaTO)

The main purpose of this ontology is connecting gamification and intelligent tutoring systems concepts. It includes representing ITS components—i.e., domain model, student model and pedagogical model—as well as their relationship with gamification concepts.

Our main sources for knowledge acquisition include the works considered in the gamification ontologies and theoretical works about ITS—i.e., the works of Du Boulay and Luckin (2001), Self (1998), de Barros Costa et al. (1998), Dillenbourg and Self (1992), and Self (1990). In fact, for the sake of making use of existing work, these works are the theoretical background of the work proposed by Bittencourt et al. (2009), which presents an integrated ITS ontology that conceptualizes ITS components according to such works.

For conceptualizing this ontology, we also define the core concepts, a glossary of terms, a tree of concepts, and binary-relations between the concepts in the ontology. Based on our sources of knowledge, we explicitly defined the following core concepts: Gamified ITS, Domain Model, Student Model, Pedagogical Model and Gamification Model.

In the integration phase of this ontology, we import the GaDO-core and GaDO-full ontologies as well as the ITS ontology provided by Bittencourt et al. (2009). Moreover, we also rely on existing RDF vocabularies—i.e., FOAF to represent personal data about students in the ontological model. We also implemented the GaTO ontology in an RDF/OWL file with the aid of Protégé tool^5^. Figure 2 presents an excerpt of this ontology integrated with GaDO-core, GaDO-full, and ITS ontologies in a UML conceptual model—the red classes represent concepts reused from the ITS ontology and the green classes represent the concepts of GaTO ontology. In the sequel, we explain each of its concepts and relations as well as how they are integrated with other ontologies.

The concepts of GaTO ontology represent the core concepts involved in a gamified intelligent tutoring system. As seen in Figure 2, besides including the three main ITS components—i.e., Student Model, Domain Model, and Pedagogical Model—a *Gamified ITS* also has a *Gamification Model*. The *Student Model* is connected to the ITS ontology through the *Behavioral Knowledge* concept, which is the representation of how a student behaves in the tutor, according to Dillenbourg and Self (1992). It is also connected to the *Player* concept of the GaDO-core ontology to include students' behaviors as players. The *Pedagogical Model* is connected to the *Instructional Plan* (Du Boulay and Luckin, 2001) concept to represent the tutoring strategies that could be used in the tutor. The *Domain Model* is, actually, a concept from the ITS ontology provided by Bittencourt et al. (2009)^6^ and is related to the *Curriculum* concept. In turn, a *Curriculum* has a set of *Resources*, also referred as learning objects. Despite been suppressed in Figure 2 for clarity purposes, these resources can be of several types, for instance, Problem, Content, Concept, Question, Essay and so on. The *Gamification Model* is connected to the *Activity Loops* designed for that gamified tutor. Furthermore, the *Action* concept from the GaDO-full ontology, which is part of a particular *Engagement Loop*, makes use of *Resources* from the ITS ontology. This relationship enables that a specific *Engagement Loop* design considers the interaction with *Resources* from an ITS *Domain Model*.

The documentation of this ontology was also produced throughout the execution of all previous phases^7^. In the next section, we also describe how we evaluate this ontology.

## 3. Ontological Model Evaluation

As previously mentioned, the METHONTOLOGY does not explicitly describe how to evaluate ontologies specified by following its steps. To evaluate our ontologies, we conduct a quantitative and qualitative evaluation with experts for each ontologies within our model.

### 3.1. Method

We used the FOCA methodology (Bandeira et al., 2016) to evaluate our ontology model. Our choice for such methodology was due because, in comparison to other ontologies evaluation strategies reported in the literature (Gruber, 1995; Gangemi et al., 1996; Gómez-Pérez, 1996; Obrst et al., 2007; Staab and Studer, 2013), this evaluation method strongly relies on the knowledge representation principles (Davis et al., 1993) as well as on constructs of other evaluation strategies to define a set of objective criteria to evaluate ontologies. The output of the evaluation is an overall quality score as well as partial scores concerned to particular knowledge representation principles, for each evaluator.

According to Bandeira et al. (2016), the ontology evaluation is performed in three steps: (1) verifying ontology's type; (2) verifying questions and metrics, and (3) computing ontology's scores. In the first step, evaluators assign the type of the ontology that is evaluated. Table 2 presents the goals, questions, and metrics that are used to ascertain ontologies' evaluation (Step 2) using the FOCA methodology. It might be worth noting that the type of the ontology enables or disables some questions of the FOCA methodology. As explained by Bandeira et al. (2016), if an ontology's type is a domain or task one, the question 4 (Q4) must not be considered for the evaluation, whereas, if it is an application type, the question 5 (Q5) is not taken into account. The overall score for an evaluator *i* is calculated by the Equation (1) on below. This same equation may be also used to calculate the partial score regarding each one of the coefficients related to the goals, for instance, to compute the score regarding the substitute goal (*Cov*_*S*_), it is only necessary to use the equation on below canceling the other coefficients (*Cov*_*Oc*_, *Cov*_*IR*_ and, *Cov*_*Ce*_).

(1) $$\begin{array}{r} {Score}_{i}=\frac{e^{\left\{-0.44+0.03{\left({Cov}_{S}\right)}_{i}+0.02{\left({Cov}_{Oc}\right)}_{i}+0.01{\left({Cov}_{Ir}\right)}_{i}+0.02{\left({Cov}_{Ce}\right)}_{i}-0.66{GExp}_{i}\right\}}}{1\;+\;e^{\left\{-0.44+0.03{\left({Cov}_{S}\right)}_{i}+0.02{\left({Cov}_{Oc}\right)}_{i}+0.01{\left({Cov}_{Ir}\right)}_{i}+0.02{\left({Cov}_{Ce}\right)}_{i}-0.66{GExp}_{i}\right\}}} \end{array}$$

Where:

- *Cov*_*S*_ is the average score for the Substitute goal's questions, including sub-questions;
- *Cov*_*Oc*_ is the average score for the Ontological commitment goal's questions – note that the ontology's type modifies the computation of this variable. If it is a task or domain ontology this variable does not take into account Q4, whereas if it is an application one, Q5 is not considered for this score;
- *Cov*_*IR*_ is the average score for the Intelligent reasoning goal's questions;
- *Cov*_*Ce*_ is the average score for the Computation efficiency goal's questions;
- *GExp* indicates the evaluator experience with the use of ontologies, if the experience is greater than 3 years, it receives 1, whereas it receives 0.

**Table 2:** Goals, questions and metrics (along with a range of possible scores) of the FOCA methodology.

**Goal**	**Question**	**Metric**	**Range of scores**
Substitute	Q1 – Are the ontology's competences defined?	Completeness	
	Q1.1 – Is there a description of the ontology's objective in the documentation?		0, 25, 50, 75, 100
	Q1.2 – Is there a description of the ontology's target public in the documentation?		0, 25, 50, 75, 100
	Q1.3 – Are there use scenarios in the documentation?		0, 25, 50, 75, 100
	Q2 – Is the ontology addressing the defined competences?		0, 25, 50, 75, 100
	Q3 – Does the ontology reuse other ontologies?	Adaptability	0, 100
Ontological commitment	Q4 – Does the ontology require a minimal knowledge commitment?	Conciseness	0, 25, 50, 75, 100
	Q5 – Does the ontology require a maximum knowledge commitment?		0, 25, 50, 75, 100
	Q6 – Are the ontology's properties coherent with the domain?	Consistency	0, 25, 50, 75, 100
Intelligent reasoning	Q7 – Are there contradictory axioms?	Consistency	0, 25, 50, 75, 100
	Q8 – Are there redundant axioms?	Conciseness	0, 25, 50, 75, 100
Computational efficiency	Q9 – Does the reasoner present modeling errors?	Computational efficiency	0, 25, 50, 75, 100
	Q10 – Does the reasoner run in a fast way?		0, 25, 50, 75, 100
Human expression	Q11 – Is documentation consistent with the modeling?	Clarity	
	Q11.1 – Are the terms presented in the ontology's documentation consistent with ontology's modeling?		0, 25, 50, 75, 100
	Q11.2 – Is there rationale and explanation of the terms presented in the ontology's documentation?		0, 25, 50, 75, 100
	Q12 – Are the concepts well-written?		0, 25, 50, 75, 100
	Q13 – Are there annotations in the ontologies defining the concepts?		0, 25, 50, 75, 100

*Adapted from Bandeira et al. (2016)*.

### 3.2. Procedure and Participants

As suggested by the FOCA methodology, the evaluation should involve the participation of human agents. Five people with experience in the use of ontologies as well as on the ontologies' domain topics—i.e., gamification and intelligent tutoring systems were selected. Among these people, four of them are from academic settings. One is an undergraduate student in Computer Science, one is a Ms.C in Computer science (which has a master thesis in the ontology topic), the last one is a Ph.D. Student—which works with gamification and ontologies in the context of computers and education, and the last one is a Ph.D. professor that has as research interests gamification, ITS, and ontologies topics. Moreover, one other participant comes from industry, and has a Ms.C in Computer Science, his thesis involved computers and education, ontology and gamification topics. All participants had prior knowledge on ontology and prior experience with the Protégé tool.

Each of the ontologies presented in this chapter used the same participants, and Table 3 shows their experience information in the topics of the ontologies as well as the settings on which the participants are inserted.

**Table 3:** Participant experience per each topic and settings.

**Participant**	**Exp. in ontologies**	**Exp. in gamification**	**Exp. in ITS**	**Settings**
P1	>3 years	<1 year	<1 year	Academic
P2	>3 years	<1 year	<1 year	Industrial
P3	>1 and <3 years	>1 and <3 years	>3 years	Academic
P4	>3 years	>3 years	>3 years	Industrial
P5	>3 years	>3 years	>3 years	Industrial

To instrument our ontological model's evaluation, for each ontology of our model (i.e., GaDO-core^8^, GaDO-full^9^, and GaTO^10^), participants were introduced to the ontologies along with their documentation through a survey. The Steps 1 and 2 of the evaluation are included in the three surveys, asking participants to assign which is the type of each ontology as well as to answer the questions presented in Table 2. We also collect from the participants their experience with ontologies, gamification, and intelligent tutoring systems as well as qualitative data about the positive and negative aspects of our ontologies.

### 3.3. Results

This section presents the analysis of the data collected in the evaluation with participants. The collected data as well as the scripts and spreadsheets used in the experimental analysis are available at https://www.dropbox.com/s/0ksrqacpfv7c8z9/Analysis.rar?dl=0. In the following section, we present the descriptive statistics of our results.

#### 3.3.1. Descriptive Statistics

**Figure 3:**
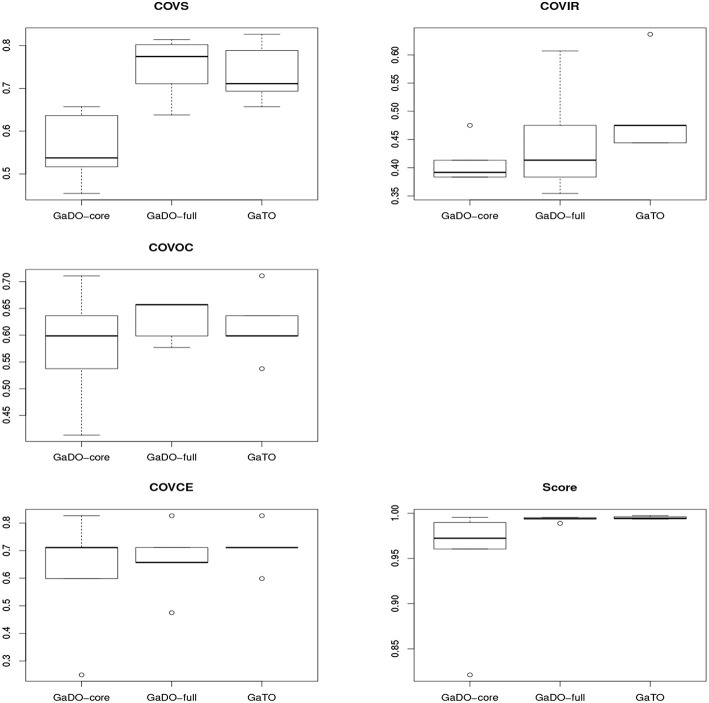
Boxplots comparing the five scores for the three ontologies.

The collected data contains the participants' answers to the questions shown in Table 2 in each one of the three ontologies. Based on those answers, we compute the ontologies' overall score as well as the score regarding the four representation knowledge goals presented in Equation (1). Thus, we conduct a descriptive analysis of the data, by analyzing histograms and boxplots of the ontologies' scores. Figure 3 presents the boxplots for each score evaluated comparing the results for the three ontologies. A brief analysis of the boxplots indicates that there is no sufficient evidence that one ontology is “better” than the others regarding the metrics considered, since there is overlap in the boxplots—the exception is the GaTO ontology compared to the GaDO-core ontology in the *Cov*_*S*_ metric, indicating that GaTO ontology has a better score. However, the hypotheses tests can confirm if there are statistical differences with significance in this metrics

We also summarize the statistics of each one of the ontologies over the five scores. Table 4 presents the summary of statistics for the *Cov*_*S*_, *Cov*_*Oc*_, *Cov*_*Ir*_, *Cov*_*Ce*_, and *Score* metrics per each ontology evaluated.

**Table 4:** Summary of statistics of the five metrics per each ontology evaluated.

**Goal**	**Ontology**	**Min**.	**1st Qu**.	**Median**	**Mean**	**3rd Qu**.	**Max**.	**Sd**.
*Cov*_*S*_	GaDO-core	0.4543	0.5167	0.5374	0.5604	0.6365	0.657	0.084877
	GaDO-full	0.638	0.7109	0.7744	0.7479	0.8022	0.8141	0.073267
	GaTO	0.657	0.6935	0.7109	0.7353	0.7886	0.8264	0.070003
*Cov*_*Oc*_	GaDO-core	0.4134	0.5374	0.5987	0.5794	0.6365	0.7109	0.112089
	GaDO-full	0.5769	0.5987	0.657	0.6293	0.657	0.657	0.038691
	GaTO	0.5374	0.5987	0.5987	0.6164	0.6365	0.7109	0.06365
*Cov*_*Ir*_	GaDO-core	0.3834	0.3834	0.3917	0.4094	0.4134	0.475	0.038673
	GaDO-full	0.3543	0.3834	0.4134	0.4466	0.475	0.6071	0.100202
	GaTO	0.444	0.444	0.475	0.4949	0.475	0.6365	0.080641
*Cov*_*Ce*_	GaDO-core	0.2497	0.5987	0.7109	0.6193	0.7109	0.8264	0.221737
	GaDO-full	0.475	0.657	0.657	0.6653	0.7109	0.8264	0.126852
	GaTO	0.5987	0.7109	0.7109	0.7116	0.7109	0.8264	0.080497
*Score*	GaDO-core	0.8213	0.9605	0.9723	0.9479	0.9897	0.9955	0.072121
	GaDO-full	0.9888	0.9935	0.994	0.9934	0.9951	0.9955	0.002672
	GaTO	0.9935	0.9937	0.9945	0.995	0.9962	0.9973	0.001637

*Cov_S_ = Substitute, Cov_Oc_ = Ontological commitment, Cov_Ir_ = Intelligent reasoning, Cov_Ce_ = Computational efficiency, and Score = Overall score*.

#### 3.3.2. Assumptions Verification and Inferential Statistics

The statistics presented are very useful to understand the overall behavior of the data regarding the scores. However, we can also analyze it to discover if there are statistically significant differences between the ontologies regarding those scores. In this way, although our intention is not discovering which ontology is better with respect to the aforementioned metrics, we compare ontologies with each other to understand if the specified ontologies have similar scores according to the FOCA methodology. Hence, we applied non-parametric tests to compare the ontologies alternatives considering the hypotheses presented in Table 5.

**Table 5:** Hypotheses of the evaluation.

H1−0: The substitute role of the ontologies is equal
H1−1: The substitute role of the ontologies is different
H2−0: The ontological commitment of the ontologies is equal
H2−1: The ontological commitment of the ontologies is different
H3−0: The intelligent reasoning of the ontologies is equal
H3−1: The intelligent reasoning of the ontologies is different
H4−0: The computational efficiency of the ontologies is equal
H4−1: The computational efficiency of the ontologies is different
H5−0: The overall scores of the ontologies are equal
H5−1: The overall scores of the ontologies are different

To verify how the ontologies' scores are compared with each other, statistical tests are applied for each one of the scores. The data of all five scores (at least for one of the ontologies) are not normal (i.e., the Shapiro–Wilk and Anderson Darling test were applied). As such, we apply a Kruskal-Wallis test to compare all three ontologies' scores and, then, we apply the Wilcoxon Test to compare the ontologies in pairs.

Table 6 presents the results of the hypotheses tests application. As shown in Table 6, the first column describes which metric is tested, the second one presents the *p*-values of the Kruskal-Wallis Test—considering as the null hypothesis that the values on all three ontologies are equal (Table 5). The third, fourth and fifth columns present the *p*-values of the ontologies' comparison, in pairs. As seen in the table, the null hypothesis for the group comparison is only rejected for the Substitute and Overall Score, respectively, with 5% and 10% of significance. Moreover, regarding the Substitute score, the null hypotheses for the comparison between GaDO-core and GaDO-full as well as between GaDO-core and GaTO are both rejected, with 5% of significance. Indeed, our results showed that the Substitute score for the GaDO-core is lower (with statistical significance) than the scores for GaDO-full and GaTO ontologies. With respect to the Intelligent Reasoning score, the null hypothesis for comparing GaDO-core and GaTO is rejected with 10% of significance, showing that the score for the GaTO ontology is better than for GaDO-core. Our tests also suggest that the null hypothesis for the comparison between the Overall Score of the GaDO-core and GaTO is rejected with 10% of significance. Concerning the Ontological Commitment and Computation Efficiency scores, our results showed that there's no statistical difference in all comparisons.

**Table 6:** *P*-values after applying Kruskal-Wallis and Wilcoxon tests (O1 = GaDO-core, O2 = GaDO-full, O3 = GaTO).

**Goal**	**μ_*O*1_ = μ_*O*2_ = μ_*O*3_**	**μ_*O*1_ = μ_*O*2_**	**μ_*O*2_ = μ_*O*3_**	**μ_*O*2_ = μ_*O*3_**
*Cov*_*S*_	0.01557**	0.01587**	0.01597**	0.9166
*Cov*_*Oc*_	0.6671	0.4578	0.7488	0.6684
*Cov*_*Ir*_	0.1552	0.8315	0.05547*	0.2888
*Cov*_*Ce*_	0.7453	1	0.6536	0.5152
*Score*	0.0977*	0.1732	0.05556*	0.4633

90% and 95% confidence levels are represented, respectively, by * and **.

### 3.4. Analysis and Discussion

In our evaluation, we also collect from the participants their comments about positive and negative aspects of our ontologies. By analyzing these comments, we can better understand what are the main reasons for the results that we have found. As previously explained, our results are only statistically significant for the Substitute and Overall Scores. Hence, we mainly focus on analyzing participants' comments aiming to explain these results.

Regarding the Substitute Score, the GaDO-core ontology received the slighter score in comparison to the other two ontologies. One participant mentioned the following statement: “I've missed some rdfs:comment in some properties in the ontology, data and object properties.”. Other two participants also mentioned that there was a lack of explanation in ontologies' properties. Another participant also states that some terms used in the ontology's descriptions are not consistent with the presented description. Two participants also commented that the ontology is not reusing any other ontology. All these comments might impact on the Substitute score since they are related to the questions Q1 and Q3. By analyzing the comments for the other two ontologies, we can observe that the comments regarding this role are less frequent. However, participants also describe a lack of annotations, been more frequent in the comment to the GaDO-core ontology.

With respect to the Overall Score, we may note that the number of participants' comments might have impacted it. Among the five participants that evaluated the GaDO-core ontology, four mentioned that there is a lack of annotations on some classes and/or properties. Two of them stated that there are problems in the definition of some classes, whereas the same number of participants also mention some confusion in the relation between some classes, for instance, between Game Design Element and Motivation and Need Theories. Moreover, two participants complained about the lack of reuse—one of them suggested to use the foaf ontology in the Player class. Finally, one participant mentioned that some terms are not consistent with classes' descriptions, and there was also one comment about problems using the reasoner. Among the participants that evaluated the GaDO-full ontology, there were two comments mentioning domain consistency problems (e.g., conceptualization using sub-classes in the Self-Determination Theory class instead of using object properties). Two participants also commented about the lack of annotations in some classes and properties, whereas there were also two comments about problems using the reasoner. Concerning the GaTO-ontology, there were also two comments mentioning the lack of annotations, one comment complaining about the lack of class definition, and one comment suggesting to improve the ontology's documentation in a general way.

Although the comments presenting some drawbacks for our ontologies, participants have also mentioned several positive aspects of them. In the GaDO-core evaluation, participants emphasized that it is easy to understand the ontology (two participants), the terms are well-written (1 participant), there is a good abstraction of the domain (1 participant), the ontology is well-designed (1 participant), and the documentation is providing a good explanation of the ontology. The comments regarding the GaDO-full include the following positive aspects: the terms are clear and well-written (2 participants), the ontology is complete (2 participants), the ontology is suitable to be applied in an educational context (1 participant), there's a good abstraction of the domain (1 participant), and there is reuse of other ontologies (1 participant). Finally, in the evaluation of the GaTO ontology, some aspects were also stressed: the terms are also well-written (1 participant), the ontology is concise (1 participant), there is a good abstraction of the domain (1 participant), there is a good level of completeness regarding the domain (1 participant) and the purpose of ontology is satisfied by connecting gamification and ITS concepts (1 participant).

Afterwards, all the aforementioned comments provided by experts were used to improve our ontologies conceptualizations.

### 3.5. Threats to Validity

This section describes concerns that must be improved in future replications of this study and other aspects that must be taken into account in order to generalize the results of the evaluation performed in this chapter. In general, the design of the evaluation aimed at minimizing a lot of the threats discussed in this section by using an objective evaluation method for ontologies (i.e., FOCA methodology). However, there are threats that should be considered. To organize this section, the threats to validity were classified using the Internal, External, Construct and Conclusion categories (Wohlin et al., 2012).

#### 3.5.1. Internal

As the experiment involves the active participation of humans, it was prone to a number of internal threats, such as (i) history—it is possible that the moment at which the experiment occurred may have affected the results, however, this threat was minimized by letting participants evaluating the ontologies at anytime they preferred; and (ii) maturation—since the participants took around 45 min to finish all the tasks of the evaluation, it is possible that they were bored or tired during the last tasks.

#### 3.5.2. Construct

The threats to the validity with respect to the construct category are closely related to the evaluation method used in the evaluation. Thus, we could not identify additional threats beyond the threats within the FOCA methodology evaluation method. However, we might be confident of this evaluation method since FOCA methodology is based on the roles for knowledge representation and all questions were validated with experts.

#### 3.5.3. External

The sample of the evaluation is representative to the academic and industrial contexts. However, the academic context is only represented by two participants and the industrial context considers only our industrial partner (i.e., MeuTutor company), thus there might be an interaction of setting and treatment threat. In fact, it is difficult to generalize the results of the experiment to other evaluators. The setting of the evaluation must be broadened to other academic and industrial settings to obtain more generic results.

#### 3.5.4. Conclusion

Furthermore, due to some restrictions, for instance, this evaluation demands participant experience in several topics (i.e., ontologies, gamification, and ITS), the sample size of the experiment was 5 participants (repetitions), thus, there might be insufficient statistical power on the effects of the evaluation. Finally, it is possible that random irrelevancies have occurred in the settings on which the participants evaluated the ontologies, e.g., noise, distractions and so on.

## 4. Intelligent Design of Gamified ITSs Based on the Ontological Model

In this section, we present an example on how to use the ontological model previously presented to develop a theory and evidence-aware authoring tool to design gamified intelligent tutoring systems. Intelligent tutoring systems design is costly and expensive (Woolf, 2010). Thus, for many years, researchers are developing ITS authoring tools in order to speed up ITS development, to reduce production efforts, to increase the number and diversity of available tutors, to extend the number of participants in ITS development process and so on (Murray, 2003; Woolf, 2010; Sottilare et al., 2015). Although the researchers' interests in the development of ITS authoring tools, the inclusion of a gamification model may require new authoring tools in order to effectively deliver gamified ITSs (Dermeval et al., 2017).

In the context of ITS design (in general), we recall a discussion about the role of human and artificial intelligence in ITS, provided by Baker (2016). He argues that tutoring systems that are currently being used at scale are much simpler than the initial vision of ITS, raising the possibility that we need “stupid tutoring systems” that are augmented with human intelligence. It means that we probably need tutors that are designed intelligently and that leverage human intelligence, rather than relying only on artificial intelligence. In this way, regarding the intelligent design of ITS – including gamified ITS – we believe that authoring tools might play an important role to achieve it effectively. Moreover, to leverage human intelligence, humans should be involved as early as possible in ITS design. Hence, a natural way to accomplish it is also relying on non-programmer authors (e.g., teachers) from the beginning of an ITS design by using authoring tools.

**Figure 4:**
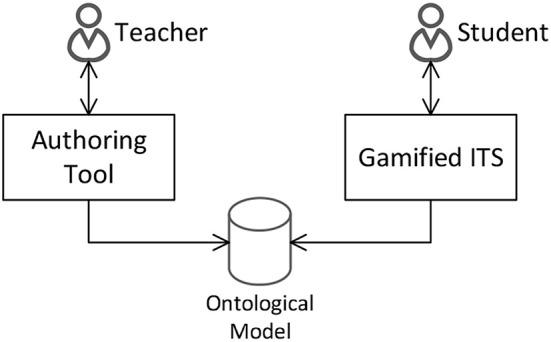
The relationship between authoring tool and gamified ITS through our ontological model.

The ontological model presented in this paper might support the development of authoring tools for intelligent designing (i.e., relying on both human and artificial intelligence) gamified ITSs. On the one hand, the design of gamified ITS could take advantage of teacher's intelligence, for instance, to define the domain model as well as to personalize the gamification model by using an ITS authoring tool. It may be worth noting that usability should be considered a high priority in the development of such a tool since teachers are the main target public. On the other hand, a gamified ITS system designed with the aid of authoring tools could strongly make use of artificial intelligence to model the behavior of students (i.e., student modeling) as well as to use suitable pedagogical strategies (i.e., pedagogical model) according to the domain and the gamification target behavior of the tutor defined by the teacher. Figure 4 illustrates the relationship between an authoring tool for teachers and a gamified ITS configured by such tool connected through our ontological model.

**Figure 5:**
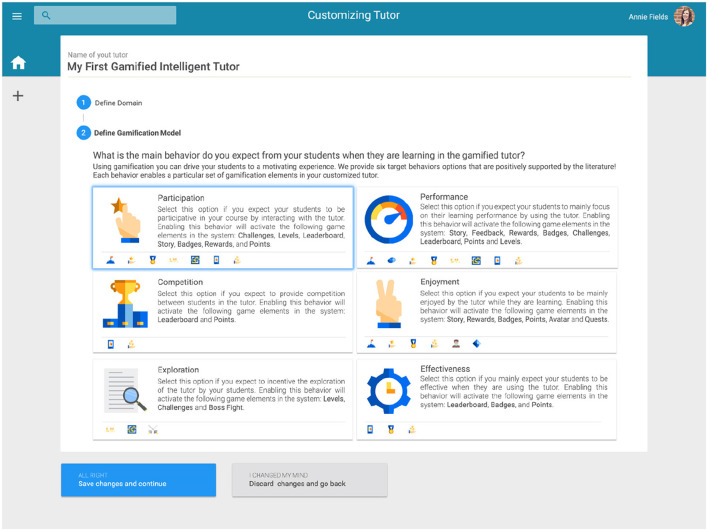
Prototype of an authoring tool illustrating the selection of a gamification target behavior.

For the authoring tool development, our ontological model might provide the knowledge representation about some gamification concepts (e.g., Self-Determination theory, BrainHex, 6D Framework) and gamification good practices in education that could be reasoned by the tool to provide a theory and evidence-aware authoring tool for teachers. This knowledge could aid the teachers in the decision-making providing them support for the authoring process. For example, Figure 5 illustrates a prototype of an authoring tool that could enable teachers to personalize the target behavior expected for their students according to the gamification good practices represented in our ontological model.

Once some components of a gamified ITS are authored by a teacher, our ontological model might work as a recipient for the authoring decision-making. Moreover, as our model is implemented using standard web technologies (i.e., OWL), the interoperability between a third-party gamified ITS and the authoring tool would occur without much effort. In this way, a gamified ITS could rely on our ontologies to reason on the components authored by teachers, e.g., domain and gamification models. It may also dynamically reason on them to select suitable tutoring strategies (i.e., following ITS theories represented in the ontologies) to use, according to the student behavior on the system for a particular domain as well as on the gamification behavior chosen by a teacher. For example, assuming that a teacher has chosen a “participation” behavior to drive the gamification design of the tutor for a math domain, as illustrated in Figure 5. A gamified ITS could make use of the ontological model to reason on the pedagogical strategies and engagement loops connected to the chosen target behavior to recommend for the students' interactions with learning objects on the domain, for instance, giving a badge after the student answers correctly five answers in geometry. It may be worth noting that, as previously mentioned, the selected target behavior constrains the game elements that could be used in the tutor, for instance, according to the literature, the participation behavior should only use challenges, levels, leaderboard, story, badges, rewards, and points. Furthermore, the behavior of students using the system as well as their player types should be also tracked and stored in the ontological model in order to be considered by the individualized tutoring of the system.

## 5. Related Works

In this work we use ontologies to conceptualize the knowledge about gamification theories and design principles to aid the application of gamification in ITS in a way that it can be automatic analyzed. Thus, as shown in Table 7, we consider four criteria to compare this work to the related works identified. In following we discuss these related works considering these criteria and Table 7 summarizes the comparison of our work to the related works.

**Table 7:** Comparison of our ontology for conceptualizing feature model and related works.

**Works**	**C1**	**C2**	**C3**	**C4**	**C5**
González et al. (2014)	Yes	No	No	No	No
Andrade et al. (2016)	Yes	Yes	Yes	Partially	No
Shi and Cristea (2016)	Yes	No	No	No	No
Challco et al. (2014)	No	Yes	No	No	Partially
Heyvaert et al. (2015)	Yes	No	No	No	Partially
Proposed model	Yes	Yes	Yes	Yes	Yes

*C1 = Apply gamification in ITS, C2=Use of gamification theories, C3 = Consider gamification evidence-supported design practices, C4=Connect gamification and ITS theories, and C5 = Formally conceptualize the knowledge*.

A conceptual architecture for building ITS considering gamification elements is proposed in González et al. (2014). The gamification elements are integrated into several modules of the system, such as game aesthetic in the student model's module and game feedbacks in the visualization module. In their work, Andrade et al. (2016) identify some problems about the use of gamification in existing gamified environments (e.g., addiction, undesired competition, and off-task behavior). For addressing such problems, they propose a framework to support the personalization of gamification for intelligent tutoring systems. An exploration of how to approach gamification in social adaptive e-learning based on the Self-Determination Theory is presented in Shi and Cristea (2016). They propose motivational gamification strategies rooted in this theory, achieving a high perceived motivation amongst students.

The aforementioned works present interesting approaches for using gamification in connection with ITS, for example, Andrade et al. (2016) explores the negative impact of gamification in learning to propose a framework for personalizing gamification, whereas Shi and Cristea (2016) achieved good effects on students' motivation using their gamification strategies. However, these works formally represent neither the knowledge about gamification theories nor the knowledge about ITS theories as well as how they are connected. In our work, we take advantage of ontologies to represented such knowledge in order to promote a more efficient reasoning and interoperability to support the development of tools that could intelligently design gamified ITS relying both on human and machine intelligence. In Andrade et al. (2016) the authors partially explore the ITS theories to apply gamification since their proposal considers some ITS components (e.g., student and tutor model). However, they do not rely on any specific ITS theories.

Ontologies have been significantly used in the domain of e-learning systems. Al-Yahya et al. (2015) present a survey of key contributions related to the development of and usage of ontologies in the e-learning domain. Their results suggest that most of the studies included in the review are using ontologies for supporting learning personalization, i.e., the main feature of ITS. However, none of these works make use of ontologies in order to support the application of gamification in ITS.

Regarding the use of ontologies for supporting the application of gamification in e-learning systems, few works are addressing such topic. Challco et al. (2014) present an ontological structure concerned with computer-supported collaborative learning (CSCL) systems to support the personalization of game design elements in collaborative learning contexts. To demonstrate its use, they show the personalization of a gamified collaborative learning scenario through a case study. However, once they target CSCL system, they only conceptualize gamification theories rather than ITS. Moreover, Heyvaert et al. (2015) present a framework that allows adding gamification to a digital textbook using standard technologies (i.e., EPUB 3 and Linked Data vocabularies). As part of their framework, they created a gamification ontology, representing some gamification concepts. This ontology is related to ours GaDO-core ontology, however, their ontology is limited to few gamification concepts (e.g., challenges, rewards, and points systems). In summary, although their contributions use ontologies for leveraging the use of gamification in the e-learning domain, they are partially targeting the use of ontologies in comparison to our proposal since none of them are using ontologies to support the application of gamification in the ITS context.

## 6. Conclusions and Future Works

Connecting gamification and ITS theories as well as providing design practices for applying gamification in ITS can contribute to the effective design of gamified ITS that take into account both learning performance and motivation of students. In this work, we connect some of these theories and define design practices for using gamification based on the literature by formally representing such concepts with the use of ontologies. Our ontological model is composed of three ontologies (i.e., GaDO-core, GaDO-full, and GaTO) and was developed following the guidelines of an ontology engineering methodology (i.e., METHONTOLOGY).

To empirically evaluate our ontological model, we used the FOCA methodology that is based on the five roles of knowledge representation. Evaluators are experts on ontologies as well as on gamification and ITS topics. The qualitative results of our ontologies' evaluation suggest that they provide a good abstraction of the domain. In addition, the results obtained with the quantitative evaluation allowed us to state: (i) there is significance on the effects of the ontology factor in the Substitute score and in the Overall score; (ii) the Substitute score of the GaDO-core ontology is lower than the scores of GaDO-full and GaTO ontologies; (iii) the Intelligent Reasoning score of the GaDO-core ontology is lower than the score of the GaTO; (iv) the Overall Score of the GaDO-core ontology is lower than the score of the GaTO ontology. (v) there is no significance on the effects of the ontology factor in the Ontological Commitment and Computational Efficiency scores; (vi) there is no statistical difference between the GaDO-full and GaTO ontologies regarding the Substitute score as well as in the Overall Score.

The results shown in this paper can be used to continually improve our ontological model to indeed support the development of intelligent authoring tools for creating gamified ITSs. As future works, we are interested in exploring authoring tools that rely both on the artificial intelligence techniques to model students' behavior and motivation, to reason on the domain knowledge, to individualize tutoring for students, and so on; as well as on the human intelligence of teachers to customize gamified ITS that take into account the context on which the tutor will be executed and teachers' pedagogical preferences.

## Author Contributions

All authors listed have made a substantial, direct and intellectual contribution to the work, and approved it for publication.

### Conflict of Interest Statement

The authors declare that the research was conducted in the absence of any commercial or financial relationships that could be construed as a potential conflict of interest.

## Footnotes

^1^The implementation of this ontology is available at https://goo.gl/qtM6Ps

^2^Available at https://goo.gl/3UKaan

^3^The implementation of this ontology is available at https://goo.gl/qtM6Ps

^4^Available at https://goo.gl/3UKaan

^5^The implementation of this ontology is available at https://goo.gl/qtM6Ps

^6^Available at https://goo.gl/qtM6Ps

^7^Available at https://goo.gl/3UKaan

^8^Evaluation form available at https://goo.gl/forms/bjKuhVp4ChCEo2ih2

^9^Evaluation form available at https://goo.gl/forms/UiF5DxJ9baCAnMLo2

^10^Evaluation form available at https://goo.gl/forms/eZYkaDYobickC7m92
